# Synthesis, Characterization and Antimicrobial Evaluation of Some New Schiff, Mannich and Acetylenic Mannich Bases Incorporating a 1,2,4-Triazole Nucleus

**DOI:** 10.3390/molecules191118897

**Published:** 2014-11-18

**Authors:** Mohamed R. Aouad

**Affiliations:** 1 Department of Chemistry, Faculty of Sciences, Taibah University, Al-Madinah Al-Munawarah 30002, Saudi Arabia; E-Mail: mr_aouad@yahoo.fr; Tel.: +966-540-953-573; 2 Laboratoire de Chimie & Electrochimie des Complexes Métalliques (LCECM) USTO-MB, Department of Chemistry, Faculty of Sciences, University of Sciences and Technology Mohamed Boudiaf, B.p.1505 El M`nouar, Oran 31000, Algeria

**Keywords:** Schiff bases, Mannich bases, acetylenic Mannich bases, 1,2,4-triazole, antimicrobial activity

## Abstract

A series of Schiff and Mannich bases derived from 4-amino-5-(3-fluoro-phenyl)-2,4-dihydro-3*H*-1,2,4-triazole-3-thione were synthesized. The alkylation of 4-phenyl-5-(3-fluorophenyl)-2,4-dihydro-3*H*-1,2,4-triazole-3-thione with propargyl bromide afforded the corresponding thiopropargylated derivative which upon treatment with the appropriate secondary amines in the presence of CuCl_2_ furnished the desired acetylenic Mannich bases. The synthesized compounds were characterized on the basis of their spectral (IR, ^1^H- and ^13^C-NMR) data and evaluated for their biological activities. Some of the compounds were found to exhibit significant antimicrobial activity.

## 1. Introduction

The 1,2,4-triazole core is considered a privileged fragment in modern heterocyclic chemistry principally due to its incorporation into a wide variety of drugs such as fluconazole, itraconazole, ribavirine, alprazolam and rizatriptan [1,2,3,4]. Moreover, some fluorinated 1,2,4-triazoles including flusilazole, fluotrimazole, epoxiconazole and flutriafol are reported to be effective fungicides [5,6]. The presence of fluorine in organic molecules often results in unexpected biological activity, which is rationalized as being due to their higher lipholicity which enhances the rate of penetration and transport of the drug to an active site [7,8]. Furthermore, the incorporation of a Schiff base moiety within the 1,2,4-triazole ring gave compounds with enhanced biological activities [9,10]. On the other hand, Mannich bases of 1,2,4-triazoles have gained importance due to their biological properties such as anticancer, antifungal, anti-inflammatory and antimalarial activities [11,12,13,14,15]. Among these, some 1,2,4-triazole Mannich bases incorporating *N*-methylpiperazine or morpholine moieties were recently found to possess antimicrobial activity [16,17,18]. In view of these facts and in an attempt to design and synthesize some novel fluorine-containing 1,2,4-triazoles with improved biological activity, a new series of Schiff, Mannich and acetylenic Mannich bases bearing fluorophenyl-1,2,4-triazole moieties were synthesized with a view to explore their potential as better antibacterial and antifungal agents.

## 2. Results and Discussion

### 2.1. *Chemistry*

**Scheme 1:**
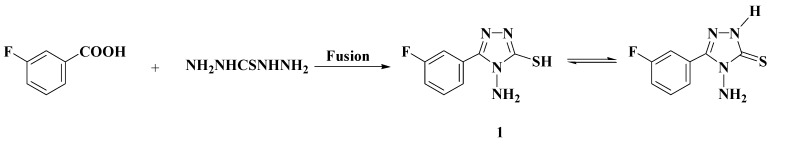
Synthesis of 4-amino-5-(3-fluorophenyl)-2,4-dihydro-3*H*-1,2,4-triazole-3-thione (**1**).

**Scheme 2:**
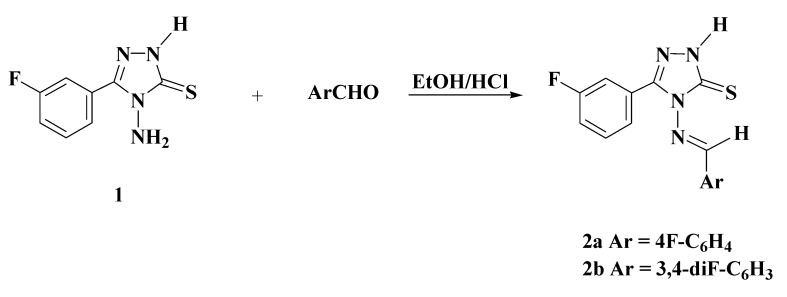
Synthesis of Schiff Bases **2a**–**2b**.

**Scheme 3:**
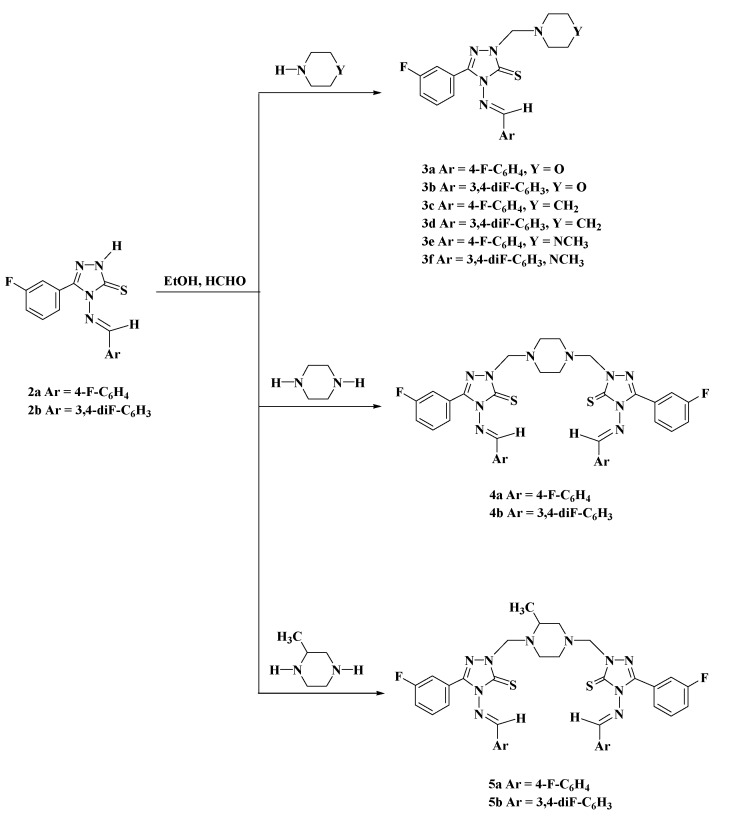
Synthesis of Mannich Bases **3a**–**3f**, **4a**–**4b**, and **5a**–**5b**.

The reaction sequences employed for the synthesis of title compounds are shown in Scheme 1, Scheme 2 and Scheme 3. The key intermediate 4-amino-5-(3-fluorophenyl)-2*H*-1,2,4-triazole-3-thione (**1**) was synthesized in 85% yield by the fusion of 3-fluorobenzoic acid with thiocarbohydrazide for 20–25 min (Scheme 1). The resulting triazole **1** was identical to that previously obtained via multi-steps synthesis [19].

The structure of the triazole **1** was confirmed by IR, ^1^H-NMR, ^13^C-NMR, and elemental analysis. In the IR spectrum of compound **1**, the NH group of the triazole ring was observed at 3227–3373 cm^−1^, thus confirming the formation of the aminotriazole. The absorption band observed at 1284 cm^−1^ could be attributed to the C=S group. The ^1^H-NMR spectrum of compound **1** showed two singlets at δ_H_ 5.82 and 13.80 ppm and a multiplet at δ_H_ 7.32–7.93 ppm corresponding to NH_2_ and NH protons, as well as the phenyl protons, respectively. The condensation of the aminotriazole **1** with 4-fluorobenzaldehyde and/or 3,4-difluorobenzaldehyde in the presence of a catalytic amount of hydrochloric acid gave Schiff bases **2a** and **2b** in good yields (Scheme 2).

All Schiff bases displayed IR, ^1^H- and ^13^C-NMR absorptions and elemental analyses consistent with the assigned structures. In the IR spectra of compounds **2a** and **2b**, the most characteristic absorptions were observed at 3288–3315 cm^−1^ (N–H), 1604–1614 cm^−1^ (C=N) and 1280–1298 cm^−1^ (C=S). Lack of resonances attributable to NH_2_ protons and the appearance of a sharp H-C=N group singlet at δ_H_ 9.79–9.89 in their ^1^H-NMR spectra agreed with the formation of Schiff bases. The ^13^C-NMR signals at δ_c_ 160.55–161.86 ppm were due to the azomethine-carbon. Moreover, the C=S group resonated at δ_C_ 164.34–165.91 ppm, thus confirming the presence of Schiff bases **2a** and **2b** in the thione form.

Mannich reactions on 3,4,5-trisubstituted-1,2,4-triazole which exist as thiol-thione tautomers gave the new Mannich bases **3a**–**3f**, **4a**–**4b**, and **5a**–**5b** via aminomethylation of the endocyclic nitrogen (N-2) of the triazole ring with formaldehyde and the appropriate secondary amine in ethanol (Scheme 3).The structural assignments of Mannich bases **3a**–**3f**, **4a**–**4b** and **5a**–**5b** were based on their elemental analysis and spectral (IR, ^1^H-NMR and ^13^C-NMR) data. In the ^1^H-NMR spectra of compounds **3a**–**3f**, the N-CH_2_-N protons resonated as singlet at δ_H_ 5.14–5.30 ppm integrating for two protons. The -CH_2_-O-CH_2_ protons corresponding to the morpholine ring resonated as a triplet at δ_H_ 3.59 ppm (*J* = 4.4 Hz) in **3a** and at 3.65 ppm (*J* = 4.6 Hz) in **3b**, while the -CH_2_-N-CH_2_- protons of the morpholine ring resonated as a triplet at δ_H_ 2.77 ppm (*J* = 4.4 Hz) and 2.82 ppm (*J* = 4.6 Hz), respectively. The methyl protons of **3e** and **3f** appeared as singlets at δ_H_ 2.12 and 2.16 ppm, respectively.

**Figure 1:**
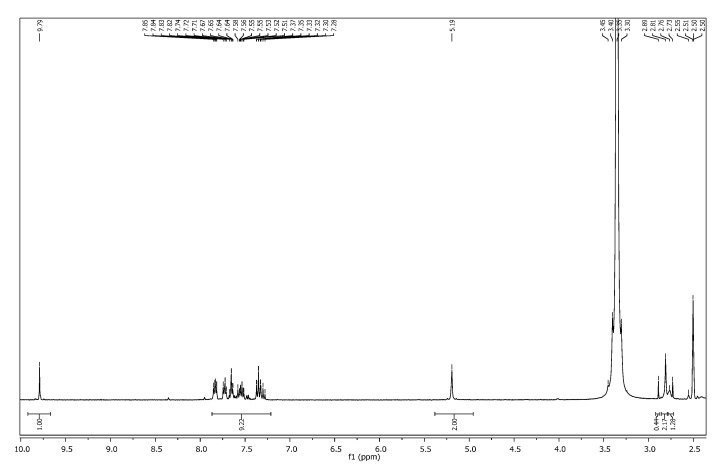
^1^H-NMR spectrum of compound **4a**.

**Figure 2:**
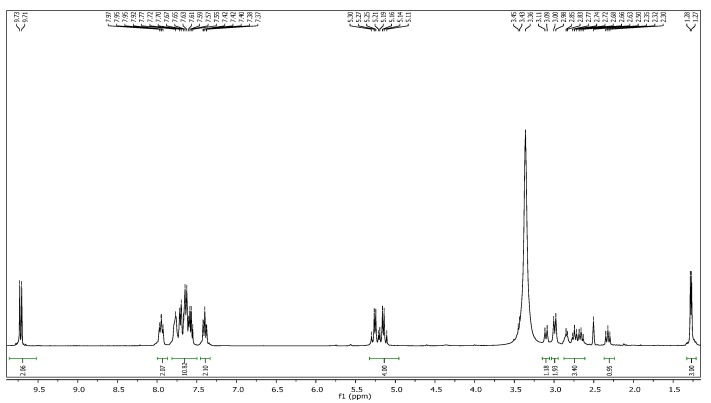
^1^H-NMR spectrum of compound **5b**.

In addition, the ^1^H-NMR spectrum of compound **4a** showed a characteristic singlet integrating for four protons at δ_H_ 5.19 ppm attributed to two N-CH_2_-N groups (Figure 1) which appeared as a multiplet at δ_H_ 5.11–5.30 in the ^1^H-NMR spectrum of compound **5b** (Figure 2).

The ^13^C-NMR spectra of Mannich bases **3a**–**3f**, **4a**–**4b**, and **5a**–**5b** showed signals at δ_c_ 160.53–166.15 and 164.89–166.64 ppm characteristic for the heterocyclic C(3) and C(5) carbons and at δ_C_ 68.60–70.21 ppm due to N-CH_2_-N.

**Scheme 4:**
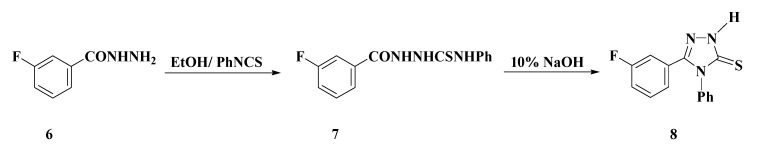
Synthesis of 5-(3-fluorophenyl)-4-phenyl-2,4-dihydro-3*H*-1,2,4-triazole-3-thione (**8**).

The commercially available 5-(3-fluorophenyl)-4-phenyl-2,4-dihydro-3*H*-1,2,4-triazole-3-thione (**8**) [CAS Registry Number: 330646-49-6] was prepared starting from 3-fluorobenzoic acid hydrazide (**6**) as outlined in Scheme 4.

**Scheme 5:**
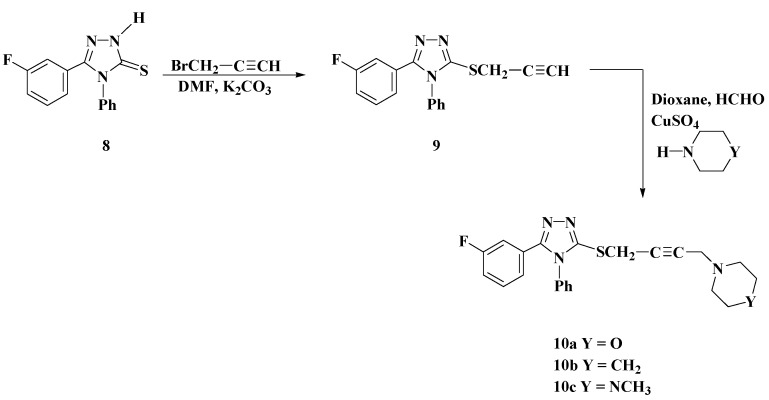
Synthesis of Acetylenic Mannich Bases **10a**–**10c**.

The reaction sequences employed for the synthesis of the acetylenic Mannich bases are shown in Scheme 5. Thus, the alkylation of compound **8** with propargyl bromide in the presence of potassium carbonate as base gave 5-(3-fluorophenyl)-4-phenyl-1,2,4-triazole-3-thio(prop-2-yne) (**9**) in good yield.

The disappearance of the NH stretch at 3345 cm^−1^ in the IR spectrum of compound **9** and appearance of the characteristic C≡C and ≡C-H bands at 2145 and 3290 cm^−1^, respectively, confirmed the formation of thiopropargylated triazole **9**. In the ^1^H-NMR spectrum of compound **9**, a triplet corresponding to the ≡C-H group was observed at δ_H_ 2.26 ppm and a doublet at δ_H_ 4.01 ppm integrating for two protons of SCH_2_ group and a peak at δ_C_ 79.85 ppm due to C≡C in the ^13^C-NMR spectrum confirmed the formation of compound **9**. The IR, ^1^H-NMR, ^13^C-NMR and elemental analysis data of compound **9** was in agreement with the assigned structure. The acetylenic Mannich bases **10a**–**10c** were synthesized in one pot multi-component Mannich reaction involving the thiopropargylated triazole **9**, formaldehyde, CuSO_4_ and the appropriate secondary amine in refluxing dioxane. The structures of the newly synthesized acetylenic Mannich bases **10a**–**10c** have been established on the basis of their elemental analysis, IR, ^1^H-NMR, and ^13^C-NMR data. The IR spectra of compounds **10a**–**10c** showed characteristic C≡C group bands at 2148–2152 cm^−1^. In addition, their ^1^H-NMR spectra showed two singlets at δ_H_ 3.43–3.46 and 4.01–4.05 ppm characteristic for C-CH_2_-N and SCH_2_ groups, respectively. The ^1^H-NMR spectrum of compound **10a** gave signals at δ_H_ 2.81 and 3.62 ppm characteristic for NCH_2_ and OCH_2_ morpholine protons, respectively.

Moreover, the ^1^H-NMR spectrum of compound **10c** gave a characteristic singlet at δ_H_ 2.24 ppm integrating for three protons of NCH_3_ group which resonated at 42.35 ppm in its ^13^C-NMR spectrum.

The ^13^C-NMR spectra of all acetylenic Mannich bases **10a**–**10c** showed characteristic signals at 22.60–22.97, 60.29–61.11 and 77.87–78.96 ppm due to SCH_2_, C-*C*H_2_-N and C≡C groups, respectively.

### 2.2. Antibacterial and Antifungal Activity

Both antimicrobial studies were assessed by minimum inhibitory concentration (MIC) assays carried out by the broth dilution method [20,21,22]. MIC is the highest dilution of a compound which shows clear fluid with no development of turbidity.

The antibacterial and antifungal screening revealed that some of the tested compounds showed good to excellent activity at 4–62.5 μg/mL in DMSO. 4-(4-Fluorobenzylideneamino)-5-(3-fluorophenyl)-2,4-dihydro-3*H*-1,2,4-triazole-3-thione (**2a**) and 4-(3,4-difluorobenzylideneamino)-5-(3-fluorophenyl)-2,4-dihydro-3*H*-1,2,4-triazole-3-thione (**2b**) showed comparatively good activity against Gram positive bacterial strains at MIC 16–31.25 μg/mL and excellent activity towards fungal strains at MIC 4–8 μg/mL. The Mannich bases **3a** and **3b** bearing a morpholine moiety showed excellent antibacterial activity against all bacterial strains and good activity against fungal species at 16–31.25 μg/mL. On the contrary, compounds **3c** and **3d** possessing a piperidine exhibited good to moderate antibacterial activity but lost the activity against the tested fungal species.

On the other hand, Mannich bases **3a**–**3f**, **4a**–**4b**, and **5a**–**5b** incorporating piperazine moieties showed excellent and greater antibacterial activity at MIC 4–16 μg/mL than antifungal action. Furthermore, 5-(3-fluorophenyl)-4-phenyl-1,2,4-triazole-3-thio(prop-2-ynyl) (**9**) exhibited moderate activity with a MIC value of 16–62.5 μg/mL. Evaluation of the antibacterial activity of the acetylenic Mannich bases **10a**–**10c**, revealed these compounds to be more effective against Gram positive bacteria at MIC 8 to 31.25 μg/mL. Particularly, acetylenic Mannich bases carrying a morpholine **10a** and/or a piperazine **10c** moiety exhibited excellent inhibition at MIC 8–16 μg/mL against Gram positive bacteria. Antifungal screening of all acetylenic Mannich bases revealed that compounds **10a** and **10c** showed excellent antifungal activity against all tested fungal strains at MIC 8–31.5 μg/mL.

The remaining compounds were found to be active at higher concentrations, e.g., 62.5 and 125 mg/mL. It was therefore concluded that the presence of a morpholine and/or piperazine moiety, in addition to 3-fluorophenyl groups, was essential for high antibacterial and antifungal activities in these compounds. The results of antibacterial and antifungal screening of the newly prepared Schiff, Mannich and acetylenic Mannich bases, expressed as MIC values, are summarized in Table 1.

**Table 1:** Antimicrobial activity expressed as MIC (μg/mL).

Compounds	Gram-Positive Organisms ^a^			Gram-Negative Organisms ^b^			Fungi ^c^	
	*Sp*	*Bs*	*Sa*	*Pa*	*Ec*	*Kp*	*Af*	*Ca*
**2a**	16	31.25	16	125	31.25	62.5	8	4
**2b**	16	16	16	31.25	62.5	31.25	4	4
**3a**	8	4	8	8	8	4	16	31.25
**3b**	8	4	4	8	4	4	16	16
**3c**	31.25	16	31.25	62.5	31.25	16	125	250
**3d**	62.5	31.25	16	16	16	16	125	250
**3e**	4	8	4	8	8	4	31.25	16
**3f**	4	8	8	8	4	8	16	16
**4a**	8	8	4	8	4	16	31.25	16
**4b**	16	4	4	8	4	8	31.25	31.25
**5a**	8	16	4	16	4	8	62.5	16
**5b**	4	16	4	8	4	16	31.25	16
**9**	16	62.5	31.25	16	16	31.25	125	250
**10a**	16	8	8	62.5	62.5	31.25	8	16
**10b**	16	31.25	31.25	31.25	125	62.5	31.25	62.5
**10c**	8	16	8	31.25	62.5	31.25	8	31.25
Ciprofloxacin	≤5	≤1	≤5	≤5	≤1	≤1	-	-
Fluconazole	-	-	-	-	-	-	≤1	≤1

Notes: ^a^: Gram-positive bacteria: *Streptococcus pneumonia* (RCMB 010010, Sp), *Bacillus subtilis* (RCMB 010067, Bs), *Staphylococcus aureus* (RCMB 010025, Sa); ^b^: Gram-negative bacteria: *Pseudomonas aeuroginosa* (RCMB 010043, Pa), *Escherichia coli* (RCMB 010052, Ec), *Klebsiella pneumonia* (RCMB 010058, Kp); ^c^: yeasts: *Aspergillus fumigatus* (RCMB 02568, Af), *Candida albicans* (RCMB 05036, Ca).

## 3. Experimental Section

### 3.1. General Information

Melting points were determined on a Melt-temp apparatus and are uncorrected. The ^1^H- and ^13^C-NMR spectra were recorded on a Bruker AC-400 NMR spectrometer operating at 400 MHz for ^1^H-NMR, 100 MHz for ^13^C-NMR. Compounds were dissolved in DMSO-d_6_ and chemical shifts were referenced to TMS (^1^H- and ^13^C-NMR). The IR spectra were measured as potassium bromide pellets using a Perkin-Elmer 1430 series FT-IR spectrometer. The elemental analyses were performed by the microanalysis unit at the Faculty of Science, Cairo University.

### 3.2. Synthesis of 4-Amino-5-(3-fluorophenyl)-2,4-dihydro-3H-1,2,4-triazole-3-thione *(**1**)*

A mixture of 3-fluorobenzoic acid (**1**, 0.01 mol) and thiocarbohydrazide (0.015 mol) was heated on a mantle in a round-bottomed flask until the contents melted. The mixture was maintained at this temperature for 15–20 min. The product obtained on cooling was treated with sodium bicarbonate solution to neutralize the unreacted carboxylic acid if any. The solid mass was then washed with water and collected by filtration. The product was recrystallized from a mixture of dimethylformamide and ethanol yielded 81% of the desired compound as white crystals: m.p. 221–222 °C, Lit m.p. 220 °C [19]. IR (*υ*, cm^−1^): 3227–3373 (NH, NH_2_), 3070 (Ar-H), 1617 (C=N), 1284 (C=S). ^1^H-NMR: δ 5.82 (s, 2H, NH_2_), 7.32–7.93 (m, 4H, ArH), 13.80 (s, 1H, NH triazole). ^13^C-NMR: δ 114.64, 114.88, 117.23, 117.44, 124.04, 127.64, 130.71, 148.23, 160.51, 162.93, 167.17 (ArC, C=N). Anal. Calcd. for C_8_H_7_FN_4_S: C 45.70, H 3.36, N 26.65. Found: C 45.92, H 3.13, N 26.40.

### 3.3. General Procedure for the Synthesis of Schiff Bases ***2a–2b***

A mixture of compound **1** (10 mmol) and the appropriate benzaldehyde derivative (10 mmol) was refluxed in ethanol (30 mL) containing HCl (1 mL) for 6 h. The solution was cooled and a yellow solid appeared. The obtained precipitate was filtered and recrystallized from ethanol to afford the desired product.

*4-(4-Fluorobenzylideneamino)-5-(3-fluorophenyl)-2,4-dihydro-3H-1,2,4-triazole-3-thione* (**2a**). This compound was obtained as colorless crystals, Yield 89%; m.p. 167–168 °C. IR (*υ*, cm^−1^): 3,288 (NH), 3,085 (Ar-H), 1,604 (C=N), 1,298 (C=S). ^1^H-NMR: δ 7.33–7.84 (m, 8H, ArH), 9.89 (s, 1H, H-C=N), 14.39 (s, 1H, NH triazole). ^13^C-NMR: δ 116.30, 116.52, 124.89, 127.41, 128.50, 128.53, 129.70, 131.00, 131.09, 132.65, 132.80, 133.21, 147.54, 161.86, 163.41, 163.93, 165.91 (ArC, C=N). Anal. Calcd. for C_15_H_10_F_2_N_4_S: C 56.95, H 3.19, N 17.71. Found: C 56.78, H 3.36, N 17.43.

*4-(3,4-Difluorobenzylideneamino)-5-(3-fluorophenyl)-2,4-dihydro-3H-1,2,4-triazole-3-thione* (**2b**). This compound was obtained as colorless crystals, Yield 87%; m.p. 195–196 °C. IR (*υ*, cm^−1^): 3,315 (NH), 3,064 (Ar-H), 1,614 (C=N), 1,280 (C=S). ^1^H-NMR: δ7.42–8.01 (m, 7H, ArH), 9.79 (s, 1H, H-C=N), 14.40 (s, 1H, NH triazole). ^13^C-NMR: δ 114.93, 115.18, 116.97, 117.15, 117.60, 117.81, 118.58, 118.75, 124.48, 124.50, 126.68, 127.24, 127.32, 129.54, 130.97, 131.05, 147.43, 151.20, 160.55, 162.45, 162.97, 164.34 (ArC, C=N). Anal. Calcd. for C_15_H_9_F_3_N_4_S: C 53.89, H 2.71, N 16.76. Found: C 54,13, H 2.50, N 16.93.

### 3.4. General Procedure for the Synthesis of Mannich Bases

A solution of Schiff base **2a** and/or **2b** (10 mmol), formaldehyde (40%, 1.5 mL) and the appropriate secondary amine (10 mmol) in ethanol (25 mL) was stirred for 2 h and left overnight at room temperature. The solid mass thus separated was collected by filtration, dried and recrystallized from ethanol/DMF.

*4-(4-Fluorobenzylideneamino)-2-(morpholin-4-ylmethyl)-5-(3-fluorophenyl)-2,4-dihydro-3H-1,2,4-triazole-3-thione* (**3a**). This compound was obtained as colorless crystals, Yield: 87%; m.p. 230–231 °C. IR (*υ*, cm^−1^): 3,046 (Ar-H), 2,826–2,971 (CH str.), 1,608 (C=N), 1,284 (C=S). ^1^H-NMR: δ 2.77 (t, 4H, *J* = 4.4 Hz, N-CH_2_), 3.59 (t, 4H, *J* = 4.4 Hz, OCH_2_), 5.21 (s, 2H, N-CH_2_-N), 7.32–7.86 (m, 8H, ArH), 9.81 (s, 1H, H-C=N). ^13^C-NMR: δ 50.32 (NCH_2_), 66.05 (OCH_2_), 69.06 (N-CH_2_-N), 116.33, 116.55, 124.48, 127.45, 128.36, 128.39, 129.75, 131.14, 131.23, 132.63, 132.97, 133.23, 146.16, 162.75, 163.52, 165.09, 166.02 (ArC, C=N). Anal. Calcd. for C_20_H_19_F_2_N_5_OS: C 57.82, H 4.61, N 16.86. Found: C 57.62, H 4.45, N 16.77.

*4-(3,4-Difluorobenzylideneamino)-2-(morpholin-4-ylmethyl)-5-(3-fluorophenyl)-2,4-dihydro-3H-1,2,4-triazole-3-thione* (**3b**). This compound was obtained as colorless crystals, Yield: 86%; m.p. 256–257 °C. IR (*υ*, cm^−1^): 3063 (Ar-H), 2842–2961 (CH str.), 1612 (C=N), 1292 (C=S). ^1^H-NMR: δ 2.82 (t, 4H, *J* = 4.6 Hz, N-CH_2_), 3.65 (t, 4H, *J* = 4.6 Hz, OCH_2_), 5.30 (s, 2H, N-CH_2_-N), 7.45–8.03 (m, 7H, ArH), 9.73 (s, 1H, H-C=N). ^13^C-NMR: δ 50.96 (NCH_2_), 68.30 (OCH_2_), 69.82 (N-CH_2_-N), 115.49, 117.23, 117.67, 117.89, 118.11, 118.48, 118.84, 124.67, 126.79, 126.90, 127.09, 127.40, 129.80, 131.22, 131.71, 145.77, 148.52, 149.50, 151.12, 151.30, 151.93, 153.67, 153.98, 160.74, 163.18, 163.69, 165.25 (ArC, C=N). Anal. Calcd. for C_20_H_18_F_3_N_5_OS: C 55.42, H 4.19, N 16.16. Found: C 58.67, H 4.34, N 16.37.

*4-(4-Fluorobenzylideneamino)-2-(piperidin-1-ylmethyl)-5-(3-fluorophenyl)-2,4-dihydro-3H-1,2,4-triazole-3-thione* (**3c**). This compound was obtained as colorless crystals solid, Yield: 85%; m.p. 204–205 °C. IR (*υ*, cm^−1^): 3082 (Ar-H), 2833–2967 (CH str.), 1600 (C=N), 1280 (C=S). ^1^H-NMR: δ 1.31–1.34 (m, 2H, CH_2_*CH_2_*CH_2_), 1.48 (t, 4H, *J* = 4.4 Hz, NCH_2_*CH_2_*), 2.74 (t, 4H, *J* = 4.4 Hz, NCH_2_), 5.18 (s, 2H, N-CH_2_-N), 7.31–7.85 (m, 8H, ArH), 9.83 (s, 1H, H-C=N). ^13^C-NMR: δ 23.43 (CH_2_*CH_2_*CH_2_), 25.48 (NCH_2_*CH_2_*), 51.20 (NCH_2_), 69.99 (N-CH_2_-N), 116.29, 116.51, 124.58, 127.42, 128.41, 128.43, 129.74, 131.10, 131.19, 132.59, 132.90, 133.25, 146.03, 162.58, 163.49, 164.78, 165.99 (ArC, C=N). Anal. Calcd. for C_21_H_21_F_2_N_5_S: C 61.00, H 5.12, N 16.94. Found: C 61.23, H 5.01, N 16.65.

*4-(3,4-Difluorobenzylideneamino)-2-(piperidin-1-ylmethyl)-5-(3-fluorophenyl)-2,4-dihydro-3H-1,2,4-triazole-3-thione* (**3d**). This compound was obtained as white solid, Yield: 85%; m.p. 237–238 °C. IR (*υ*, cm^−1^): 3,095 (Ar-H), 2,847–2,983 (CH str.), 1,622 (C=N), 1,299 (C=S). ^1^H-NMR: δ 1.29–1.33 (m, 2H, CH_2_*CH_2_*CH_2_), 1.49 (t, 4H, *J* = 4.5 Hz, NCH_2_*CH_2_*), 2.73 (t, 4H, *J* = 4.5 Hz, NCH_2_), 5.14 (s, 2H, N-CH_2_-N), 7.41–8.01 (m, 7H, ArH), 9.76 (s, 1H, H-C=N). ^13^C-NMR: δ 23.33 (CH_2_*CH_2_*CH_2_), 25.47 (NCH_2_*CH_2_*), 51.13 (NCH_2_), 70.21 (N-CH_2_-N), 115.27, 116.98, 117.17, 117.72, 117.93, 118.52, 118.70, 124.59, 126.69, 126.74, 126.86, 126.95, 129.44, 130.97, 131.05, 145.98, 148.60, 148.73, 150.96, 151.07, 151.20, 153.49, 153.61, 160.54, 162.96, 163.19, 164.89 (ArC, C=N). Anal. Calcd. for C_21_H_20_F_3_N_5_S: C 58.46, H 4.67, N 16.23. Found: C 58.17, H 4.81, N 16.41.

*4-(4-Fluorobenzylideneamino)-5-(3-fluorophenyl)-2-[(4-methylpiperazin-1-yl)methyl]-2,4-dihydro-3H-1,2,4-triazole-3-thione* (**3e**). This compound was obtained as white solid, Yield: 84%; m.p. 191–192 °C. IR (*υ*, cm^−1^): 3,061 (Ar-H), 2838–2978 (CH str.), 1604 (C=N), 1290 (C=S). ^1^H-NMR: δ 2.12 (s, 3H, NCH_3_), 2.78 (t, 4H, *J* = 4.6 Hz, NCH_2_), 3.31 (t, 4H, *J* = 4.6 Hz, NCH_2_), 5.18 (s, 2H, N-CH_2_-N), 7.40–8.02 (m, 8H, ArH), 9.68 (s, 1H, H-C=N). ^13^C-NMR: δ 45.72 (NCH_3_), 49.69 (NCH_2_), 54.51 (NCH_2_), 69.14 (N-CH_2_-N), 115.03, 115.27, 116.41, 116.63, 117.74, 117.95, 124.56, 124.59, 126.94, 127.03, 128.35, 128.37, 131.00, 131.08, 131.37, 131.46, 146.01, 146.04, 160.53, 162.96, 163.33, 163.64, 166.15, 166.64 (ArC, C=N). Anal. Calcd. for C_21_H_22_F_2_N_6_S: C 58.86, H 5.17, N 19.61. Found: C 59.10, H 5.32, N 19.46.

*4-(3,4-Difluorobenzylideneamino)-5-(3-fluorophenyl)-2-[(4-methylpiperazin-1-yl)methyl]-2,4-dihydro-3H-1,2,4-triazole-3-thione* (**3f**). This compound was obtained as white solid, Yield: 82%; m.p. 224–225 °C. IR (*υ*, cm^−1^): 3042 (Ar-H), 2830–2989 (CH str.), 1612 (C=N), 1288 (C=S). ^1^H-NMR: δ 2.16 (s, 3H, NCH_3_), 2.36 (t, 4H, *J* = 4.6 Hz, NCH_2_), 2.79 (t, 4H, *J* = 4.6 Hz, NCH_2_), 5.19 (s, 2H, N-CH_2_-N), 7.39–8.05 (m, 7H, ArH), 9.74 (s, 1H, H-C=N). ^13^C-NMR: δ 45.61 (NCH_3_), 49.59 (NCH_2_), 54.44 (NCH_2_), 69.11 (N-CH_2_-N), 114.81, 115.05, 115.11, 115.36, 116.89, 117.07, 117.37, 117.58, 117.83, 118.04, 118.55, 118.60, 118.72, 118.78, 124.37, 124.68, 126.83, 126.92, 127.50, 130.91, 131.06, 131.14, 146.15, 147.44, 160.56, 162.55, 162.97, 163.24, 163.73, 165.35 (ArC, C=N). Anal. Calcd. for C_21_H_21_F_3_N_6_S: C 56.49, H 4.74, N 18.82. Found: C 56.71, H 4.89, N 18.59.

*1,4-Bis{[4-(4-fluorobenzylideneamino)-5-(3-fluorophenyl)-2,4-dihydro-3H-1,2,4-triazole-3-thione]-2-methyl}piperazine* (**4a**). This compound was obtained as colorless crystals, Yield: 81%; m.p. 263–264 °C. IR (*υ*, cm^−1^): 3088 (Ar-H), 2827–2965 (CH str.), 1611 (C=N), 1296 (C=S). ^1^H-NMR: δ 2.73 (t, 2H, *J* = 4.8 Hz, NCH_2_), 2.76 (bs, 2H, NCH_2_), 2.81 (t, 3H, *J* = 4.8 Hz, NCH_2_), 2.89 (t, 1H, *J* = 4.8 Hz, NCH_2_), 5.19 (s, 4H, N-CH_2_-N), 7.28–7.85 (m, 16H, ArH), 9.79 (s, 2H, 2 × H-C=N). ^13^C-NMR: δ 51.57 (NCH_2_), 69.42 (N-CH_2_-N), 115.22, 115.51, 116.33, 116.94, 117.41, 118.72, 124.79, 125.68, 126.79, 127.90, 128.43, 131.46, 131.64, 131.81, 132.19, 146.20, 146.33, 163.56, 164.02, 165.49, 166.36 (ArC, C=N). Anal. Calcd. for C_36_H_30_F_4_N_10_S_2_: C 58.21, H 4.07, N 18.86. Found: C 58.40, H 4.26, N 18.61.

*1,4-Bis{[4-(3,4-difluorobenzylideneamino)-5-(3-fluorophenyl)-2,4-dihydro-3H-1,2,4-triazole-3-thione]-2-methyl}piperazine* (**4b**). This compound was obtained as colorless crystals, Yield: 80%; m.p. 277–278 °C. IR (*υ*, cm^−1^): 3067 (Ar-H), 2840–2969 (CH str.), 1603 (C=N), 1291 (C=S). ^1^H-NMR: δ 2.73 (t, 2H, *J* = 4.4 Hz, NCH_2_), 2.78 (bs, 2H, NCH_2_), 2.82 (t, 3H, *J* = 4.4 Hz, NCH_2_), 2.89 (t, 1H, *J* = 4.4 Hz, NCH_2_), 5.17 (s, 4H, N-CH_2_-N), 7.42–8.00 (m, 14H, ArH), 9.71 (s, 2H, 2 × H-C=N); ^13^C-NMR: δ 51.78 (NCH_2_), 69.90 (N-CH_2_-N), 114.65, 115.19, 115.35, 115.90, 117.08, 117.15, 117.69, 117.89, 118.22, 118.70, 118.97, 119.26, 125.80, 126.74, 126.95, 127.17, 127.83, 130.57, 131.43, 131.60, 146.42, 147.92, 161.34, 162.42, 162.84, 163.50, 164.88, 165.04 (ArC, C=N). Anal. Calcd. for C_36_H_28_F_6_N_10_S_2_: C 55.52; H 3.62, N 17.99. Found: C 55.78, H 3.49, N 17.73.

*1,4-Bis{[4-(4-fluorobenzylideneamino)-5-(3-fluorophenyl)-2,4-dihydro-3H-1,2,4-triazole-3-thione]-2-methyl}-2-methylpiperazine* (**5a**). This compound was obtained as colorless crystals, Yield: 78%; m.p. 257–258 °C. IR (*υ*, cm^−1^): 3,084 (Ar-H), 2,841–2,973 (CH str.), 1,606 (C=N), 1,293 (C=S). ^1^H-NMR: δ 1.29 (d, 3H, *J* = 4.0 Hz, CH_3_), 2,13 (t, 1H, *J* = 4.0 Hz, NCH_2_), 2.66–2.89 (m, 3H, C*H*CH_3_, NCH_2_), 3.02 (d, 2H, *J* = 4.0 Hz, NCH_2_), 3.09 (d, 1H, *J* = 4.0 Hz, NCH_2_), 5.15–5.31 (m, 4H, N-CH_2_-N), 7.42–78.00 (m, 16H, ArH), 9.63, 9.66 (2s, 2H, 2 × H-C=N). ^13^C-NMR: δ 28.85 (NCH_3_), 49.26, 51.72, 51.96 (NCH_2_), 69.17, 69.73 (N-CH_2_-N), 115.12, 115.48, 115.70, 116.42, 117.43, 117.63, 118.05, 118.30, 118.96, 119.22, 119.54, 124.82, 125.74, 126.39, 127.18, 127.79, 129.14, 130.45, 130.78, 131.33, 132.50, 145.42, 146.36, 148.70, 161.45, 162.00, 162.52, 163.70, 164.47, 165.55 (ArC, C=N). Anal. Calcd. for C_37_H_32_F_4_N_10_S_2_: C 58.72, H 4.26, N 18.51. Found: C 58.48, H 4.06, N 18.76.

*1,4-Bis{[{[4-(3,4-difluorobenzylideneamino)-5-(3-fluorophenyl)-2,4-dihydro-3H-1,2,4-triazole-3-thione]-2-methyl}-2-methylpiperazine* (**5b**). This compound was obtained as white solid, Yield: 76%; m.p. 284–285 °C. IR (υ, cm^−1^): 3,070 (Ar-H), 2,842–2,982 (CH str.), 1,602 (C=N), 1,295 (C=S). ^1^H-NMR: δ 1.28 (d, 3H, *J =* 4.0 Hz, CH_3_), 2,32 (t, 1H, *J* = 4.0 Hz, NCH_2_), 2.63–2.85 (m, 3H, CHCH_3_, NCH_2_), 3.00 (d, 2H, *J =* 4 Hz, NCH_2_), 3.11 (d, 1H, *J =* 4 Hz, NCH_2_), 5.11–5.30 (m, 4H, N-CH_2_-N), 7.40–7.95 (m, 14H, ArH), 9.71, 9.73 (2s, 2H, H-C=N). ^13^C-NMR: δ 28.66 (NCH_3_), 49.43, 50.98, 51.48 (NCH_2_), 68.60, 69.04 (N-CH_2_-N), 113.88, 114.79, 115.48, 116.11, 117.27, 117.34, 117.84, 118.15, 118.50, 118.86, 119.24, 119.58, 119.90, 125.96, 126.56, 126.98, 127.30, 128.23, 129.66, 130.83, 131.57, 131.88, 132.77, 145.65, 146.55, 148.30, 161.61, 162.12, 162.76, 162.89, 163.62, 164.62, 165.32, 166.58 (ArC, C=N). Anal. Calcd. for C_37_H_30_F_6_N_10_S_2_: C 56.05, H 3.81, N 17.67. Found: C 55.87, H 3.67, N 17.91.

### 3.5. Synthesis of 5-(3-Fluorophenyl)-4-phenyl-2,4-dihydro-3H-1,2,4-triazole-3-thione *(**8**)*

A mixture of compound **7** (10 mmol) and 10% sodium hydroxide solution (100 mL) was refluxed for 4 h. The mixture was then cooled to room temperature and filtered. The filtrate was acidified by the addition of hydrochloric acid. The resulting solid was collected by filtration, dried and recrystallized from ethanol yielded 83% of **8** as white solid; m.p. 175–176 °C. IR (*υ*, cm^−1^): 3,345 (N-H), 3,095 (Ar-H), 1,619 (C=N), 1,289 (C=S). ^1^H-NMR: δ 7.03–7.56 (m, 9H, Ar-H), 14.22 (s, 1H, NH). ^13^C-NMR: δ 115.00, 115.24, 117.22, 117.43, 124.49, 127.80, 127.89, 128.70, 129.35, 129.53, 130.75, 130.83, 134.28, 149.34, 160.27, 162.70, 168.74 (ArC, C=N). Anal. Calcd. for C_14_H_10_FN_3_S: C 61.98, H 3.72, N 15.49. Found: C 61.70, H 3.51, N 15.77.

### 3.6. Synthesis of 5-(3-Fluorophenyl)-4-phenyl-1,2,4-triazole-3-thio(prop-2-yne) *(**9**)*

To a stirred solution of compound **8** (10 mmol) and triethylamine (10 mmol) in ethanol (25 mL), was added propargyl bromide (10 mmol) dropwise. The mixture was refluxed for one hour. Excess ethanol was removed *in vacuo*. The product was collected and crystallized from ethanol yielded 90% of **9** as white solid; m.p. 148–149 °C. IR (*υ*, cm^−1^): 3290 (≡C-H), 3058 (Ar-H), 2854–2984 (CH str.), 2145 (C≡C), 1,623 (C=N). ^1^H-NMR: δ 2.26 (t, 1H, *J =* 4.5 Hz, ≡CH), 4.01 (d, 2H, *J =* 4.5 Hz, SCH_2_), 7.15–7.60 (m, 9H, Ar-H). ^13^C-NMR: δ 21.30 (SCH_2_), 75.15 (≡CH), 79.85 (C≡C), 115.08, 115.24, 117.21, 117.35, 124.56, 124.58, 128.19, 129.07, 129.13, 130.52, 130.80, 131.29, 131.35, 133.93, 151.32, 153.95, 161.30, 162.91 (ArC, C=N). Anal. Calcd. for C_17_H_12_FN_3_S; C 66.00, H 3.91, N 13.58. Found: C 66.17, H 3.72, N 13.74.

### 3.7. General Procedure for Preparation of Acetylenic Mannich Bases ***10a–10c***

To a stirring solution of compound **9** (5 mmol) in dioxane (25 mL) was added cuprous chloride (0.0025 g) and the mixture was heated for a few min, then paraformaldehyde (5 mmol) and the appropriate secondary amine (5 mmol) were added. The mixture was heated at 90 °C for four h. After cooling, the mixture was filtered then poured onto ice water (100 mL). The residue was extracted with chloroform (3 × 25 mL) and purified on a column of silica gel using ethylacetate-hexane (1:3).

*1-{4-[5-(3-Fluorophenyl)-4-phenyl-1,2,4-triazol-3-ylthio]but-2-ynyl}-morpholine* (**10a**). This compound was obtained as white solid, Yield: 81%; m.p. 190–191 °C. IR (*υ*, cm^−1^): 3,046 (Ar-H), 2829–2980 (CH str.), 2148 (C≡C), 1607 (C=N). ^1^H-NMR: δ 2.81 (t, 4H, *J* = 4.6 Hz, N-CH_2_), 3.44 (s, 2H, ≡C-CH_2_-N), 3.62 (t, 4H, *J* = 4.6 Hz, OCH_2_), 4.05 (s, 2H, SCH_2_), 7.10–7.53 (m, 9H, ArH). ^13^C-NMR: δ 22.78 (SCH_2_), 53.46 (NCH_2_), 60.29 (≡C-CH_2_-N), 78.96 (C≡C), 115.17, 115.41, 117.45, 117.66, 124.69, 124.72, 127.37, 127.46, 128.73, 129.39, 129.67, 130.82, 130.90, 134.75, 147.94, 160.24, 162.67, 169.71 (ArC, C=N). Anal. Calcd. for C_22_H_21_FN_4_OS: C 64.69, H 5.18, N 13.72. Found: C 64.93, H 5.01, N 13.54.

*1-{4-[5-(3-Fluorophenyl)-4-phenyl-1,2,4-triazol-3-ylthio]but-2-ynyl}-piperidine* (**10b**). This compound was obtained as white solid, Yield: 80%; m.p. 221–222 °C. IR (*υ*, cm^−1^): 3037 (Ar-H), 2828–2980 (CH str.), 2152 (C≡C), 1613 (C=N). ^1^H-NMR: δ 1.38–1.42 (m, 2H, CH_2_*CH_2_*CH_2_), 1.60 (t, 4H, *J* = 5.0 Hz, NCH_2_*CH_2_*), 2.68 (t, 4H, *J* = 5.0 Hz, NCH_2_), 3.46 (s, 2H, ≡C-CH_2_-N), 4.02 (s, 2H, SCH_2_), 7.08–7.53 (m, 9H, ArH). ^13^C-NMR: δ 22.60 (SCH_2_), 23.37 (CH_2_*CH_2_*CH_2_), 25.48 (NCH_2_*CH_2_*), 51.13 (NCH_2_), 60.76 (≡C-CH_2_-N), 77.87 (C≡C), 115.09, 115.33, 117.42, 117.63, 124.62, 124.65, 127.39, 127.48, 128.71, 129.40, 129.65, 130.84, 130.92, 134.77, 147.74, 160.26, 162.69, 169.57 (ArC, C=N). Anal. Calcd. for C_23_H_23_FN_4_S: C 67.95, H 5.70, N 13.78. Found: C 67.67, H 5.98, N 13.62.

*1-{4-[5-(3-Fluorophenyl)-4-phenyl-1,2,4-triazol-3-ylthio]but-2-ynyl}-4-methylpiperazine* (**10c**). This compound was obtained as white solid, Yield: 78%; m.p. 173–174 °C. IR (*υ*, cm^−1^): 3,061 (Ar-H), 2836–2960 (CH str.), 2149 (C≡C), 1,603 (C=N). ^1^H-NMR: δ (s, 3H, NCH_3_), 2.44 (t, 4H, *J* = 5.0 Hz, NCH_2_), 2.63 (t, 4H, *J* = 5.0 Hz, NCH_2_), 3.43 (s, 2H, ≡C-CH_2_-N), 4.01 (s, 2H, SCH_2_), 7.10–7.56 (m, 9H, ArH). ^13^C-NMR: δ 22.97 (SCH_2_), 42.35 (NCH_3_), 51.32 (NCH_2_), 56.62 (NCH_2_), 61.11 (≡C-CH_2_-N), 78.16 (C≡C), 115.29, 115.54, 117.80, 118.27, 124.46, 124.95, 127.90, 128.26, 128.54, 129.16, 129.80, 131.36, 131.52, 134.63, 147.64, 160.42, 162.80, 169.98 (ArC, C=N). Anal. Calcd. for C_23_H_24_FN_5_S: C 65.53, H 5.74, N 16.61. Found: C 65.42, H 5.48, N 16.85.

### 3.8. Biological Assays

#### 3.8.1. Cells

The newly synthesized compounds **2a**–**2b**, **3a**–**3f**, **4a**–**4b**, **5a**–**5b**, **9** and **10a**–**10c** were tested for their *in vitro* growth inhibitory activity against the standard pathogenic strains of the Regional Center for Mycology and Biotechnology (RCMB) namely; *Streptococcus pneumonia* RCMB 010010, *Bacillus subtilis* RCMB 010067, *Staphylococcus aureus* RCMB 010025 (Gram-positive bacteria), *Pseudomonas aeuroginosa* RCMB 010043, *Escherichia coli* RCMB 010052, *Klebsiella pneumonia* RCMB 010058 (Gram-negative bacteria), and the yeast-like pathogenic fungus *Aspergillus fumigatus* RCMB 02568 and *Candida albicans* RCMB 05036.

#### 3.8.2. *Antibacterial and Antifungal Assays*

Preliminary antimicrobial activities of the newly synthesized compounds **2a**–**2b**, **3a**–**3f**, **4a**–**4b**, **5a**–**5b**, **9** and **10a-**were tested by broth microdilution method [20,21,22]. The MIC determination of the synthesized compounds was carried out in side-by-side comparison with ciprofloxacin against Gram-positive bacteria (*S. pneumonia*, *B. subtilis*, *S. aureus*) and Gram-negative (*P. aeruginosa*, *E. coli*, *K. penumonia*). The antifungal activity was assayed against yeasts (*A. fumigatus*, *C. albicans*). The minimum inhibitory concentrations of the compounds were recorded as the lowest concentration of each chemical compounds in the tubes with no turbidity (*i.e*., no growth) of inoculated bacteria/fungi. Test compounds (10 mg) were dissolved in dimethylsulfoxide (DMSO, 1 mL) then diluted in culture medium (Mueller-Hinton Broth for bacteria and Sabouraud Liquid Medium for fungi), further progressive dilutions to obtain final concentrations of 1, 2, 4, 8, 16, 31.25, 62.5, 125, 250 and 500 mg/mL. DMSO never exceeded 1% v/v. The tubes were inoculated with 105 cfu∙mL^−1^ (colony forming unit/mL) and incubated at 37 °C for 24 h. The growth control consisting of media and media with DMSO at the same dilutions as used in the experiments was employed.

## 4. Conclusions

New Schiff, Mannich and acetylenic Mannich bases containing 1,2,4-triazole and fluorophenyl moieties were successfully synthesized. Antimicrobial activity screening revealed that some of the tested compounds exhibited good antibacterial and antifungal activities. The combination of three biologically potent units, namely Schiff base, morpholine/piperazine and 1,2,4-triazole in one framework is essential for significant antimicrobial activity.
